# The effect of various heights of high-heeled shoes on foot arch deformation: Finite element analysis

**DOI:** 10.1186/1757-1146-7-S1-A78

**Published:** 2014-04-08

**Authors:** Amir Ahmady, Ehsan Soodmand, Iman Soodmand, Thomas L Milani

**Affiliations:** 1 Department of Biomedical Engineering, University of Malaya, Kuala Lumpur, Malaysia; 2 Technische Universität Chemnitz, Chemnitz, Germany; 3 Department of Mechanical Engineering, Jondishapour University of Technology, Dezful, Iran

## 

Women are interested to wear high-heeled shoes to increase their attractiveness. High-heeled shoes might create harmful effects on the musculoskeletal system. Besides earlier studies proved that the function of foot and lower extremity will change due to wearing high-heeled shoes [1,2]. Because of limitations of the experimental methods, direct measurements of internal strains and stresses of the foot are inconceivable or invasive [3]. In this research the comparison of the effect of 3 different sizes of height of high-heeled shoes on foot bones and plantar fascia is the main objective. Von-Misses stresses, strain, and arch deformation of the foot during balanced standing in women are the parameters which are investigated in this research. The output of this research is describing the effect of increasing of heel height on foot bones stresses.

Mimics and ABAQUS software are employed to create a finite element (FE) model of the human ankle. MIMICS used as the segmentation software and ABAQUS used for finite element analysis (FEA). A CT (Computed Topography) scan images from the right foot of a normal female subject was imported into MIMICS. The segmented surfaces were then imported into SolidWorks CAD (Computer aided design) system to create model assembly. In order to creating tetrahedral finite element meshes the solid models of foot bones and encapsulated soft tissue structures models established in MIMICS software is imported into ABAQUS. Contact interactions among the major joints were prescribed to allow relative bone movements.

The soft tissue and orthotic material were defined as hyper elastic while other tissues were idealized as homogeneous, isotropic, and linearly elastic. The ground reaction and extrinsic muscles forces for simulating the stance phase of gait were applied at the inferior ground support as a boundary condition and at their corresponding points of insertion by defining contraction forces via axial connector elements. During the balance standing condition, on half of body weight is transferred from each foot to the ground [4].

**Figure 1:**
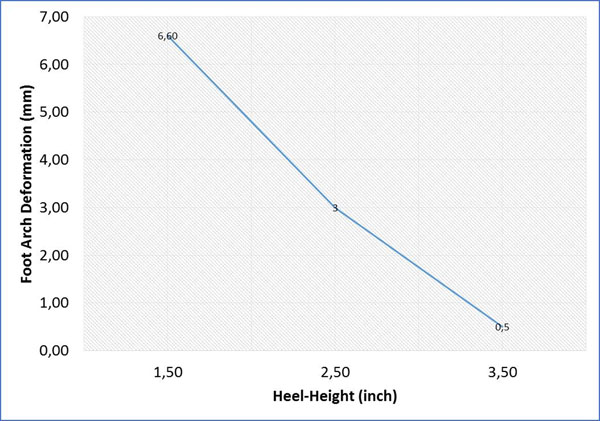
Foot arch deformations during balanced standing

**Figure 2:**
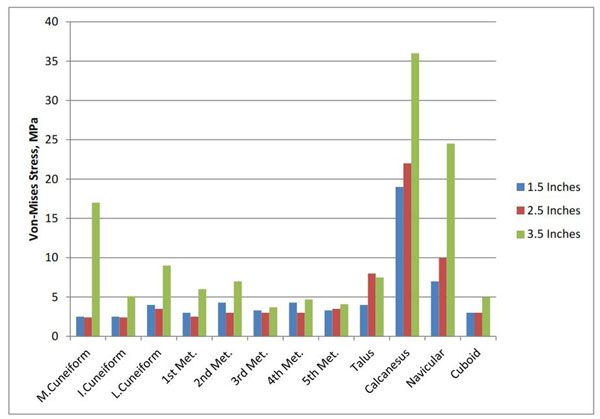
Von-Mises stress of foot bones for 3 different sizes of shoe heel heights

**Figure 3:**
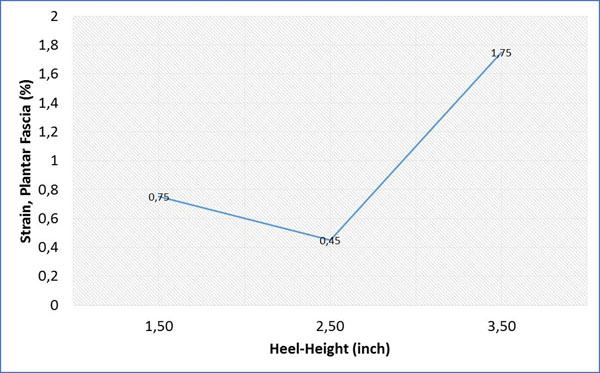
Average of strain in plantar fascia

The result of this study on the shoes with heel height 1.5 inches, 2.5 inches, and 3.5 inches shows that an increase in shoe heel height resulted in a decrease in arch deformation (Figure 1). There was a common rise in a peak Von-Mises stress of foot bones with increasing shoe heel height (Figure 2). With 2.5 inches high-heeled shoe, the strain and the total tensional force in the plantar fascia was minimum (Figure 3).
